# Supplementary material for: Incoherent non-Hermitian skin effect in photonic quantum walks
Source: Light Sci Appl. 2024 Apr 25;13:95. doi: 10.1038/s41377-024-01438-w (PMC11043335; doi:10.1038/s41377-024-01438-w)
Supplement: Supplementary file 1 — Supplementary Material [file 41377_2024_1438_MOESM1_ESM.pdf]

INCOHERENT NON-HERMITIAN SKIN EFFECT IN PHOTONIC QUANTUM WALKS
SUPPLEMENTARY MATERIAL

Stefano Longhi
Dipartimento di Fisica, Politecnico di Milano, Piazza Leonardo da Vinci 32, 20133, Milano, Italy
IFISC (UIB-CSIC), Instituto de Fisica Interdisciplinar y Sistemas Complejos - Palma de Mallorca, Spain

In this Supplemental Material, we provide some technical details and analytical derivations of results presented in the main manuscript.

S1. Derivation of the Markov transition matrix

Under fully coherent dynamics, the wave function of the system evolves according to $|\psi(t)\rangle = \hat{U}_{coh}(t)|\psi(0)\rangle$, where $\hat{U}_{coh}(t) = \exp(-i\hat{H}t)$ is the coherent propagator. Normalization of the wave function at each time instant, i.e. $|\psi(t)\rangle \rightarrow |\psi(t)\rangle / \| \psi(t) \|$, can be assumed. The incoherent dynamics is obtained as a dephasing process by assuming that, at the time instants $t_\alpha = \alpha \Delta t^+ = \Delta t^+, 2\Delta t^+, 3\Delta t^+, ...$ spaced by the time interval Δt, the phase of the wave function amplitude $\psi_n(t_\alpha) = \langle n | \psi(t_\alpha) \rangle$ is randomized, i.e. it is multiplied by a random phase $\phi_n^{(\alpha)}$, with $\phi_n^{(\alpha)}$ uncorrelated in both site index n and time step α. After letting $\rho_{n,m}(t) = \overline{\psi_n^*(t)\psi_m(t)}$, where the overbar denotes statistical average over the random phase distribution, after each time t_α one clearly has $\rho_{n,m} = 0$ for $n \neq m$, i.e. we have classicalization of the dynamics, which is fully described by a discrete-time map for the unnormalized occupation probabilities $P_n(t) = \rho_{n,n}(t)$ of the various lattice sites. To calculate the evolution of $P_n(t)$, let us indicate by $S_{n,l} = (U_{coh}(\Delta t))_{n,l} = \{\exp(-iH\Delta t)\}_{n,l}$ the matrix elements of the coherent evolution propagator over the coherence time interval Δt, and let $\phi_n^{(\alpha)}$ be the stochastic phase impressed at lattice site n and at time $t_\alpha = \alpha \Delta t^+$. Taking into account that

$$\psi_n(t_\alpha) = \exp(i\phi_n^{(\alpha)}) \sum_{l=1}^{N} S_{n,l} \psi_l(t_{\alpha-1}) \tag{S1}$$

by recursion one obtains

$$\psi_n(t_\alpha) = \sum_{l_1, l_2, ..., l_\alpha} \exp(i\phi_n^{(\alpha)} + i\phi_{l_1}^{(\alpha-1)} + ... + i\phi_{l_{\alpha-1}}^{(1)}) \times S_{n,l_1} \times S_{l_1,l_2} \times ... \times S_{l_{\alpha-1},l_\alpha} \psi_{l_\alpha}(t=0) \tag{S2}$$

where each index l_1, l_2,.., l_α in the sum varies from 1 to N. From the above equation one has

$$|\psi_n(t_\alpha)|^2 = \sum_{\sigma_1, l_1, \sigma_2, l_2, ..., \sigma_\alpha, l_\alpha} \exp(i\phi_{l_1}^{(\alpha-1)} - i\phi_{\sigma_1}^{(\alpha-1)} + ... + i\phi_{l_{\alpha-1}}^{(1)} - i\phi_{\sigma_{\alpha-1}}^{(1)}) \times S_{n,l_1} \times S^*_{n,\sigma_1} \times$$
$$\times S_{l_1,l_2} \times S^*_{\sigma_1,\sigma_2} \times ... \times S_{l_{\alpha-1},l_\alpha} S^*_{\sigma_{\alpha-1},\sigma_\alpha} \psi_{l_\alpha}(t=0) \psi^*_{\sigma_\alpha}(t=0) \tag{S3}$$

The (unnormalized) probability $P_n(t)$ that at time $t = t_\alpha = \alpha \Delta t^+$ the excitation occupies the n-th site of the lattice is obtained from the above expression after statistical average over the different realizations of stochastic phases, i.e.

$$P_n(t) = \overline{|\psi_n(t)|^2}. \tag{S4}$$

Since the phases are uncorrelated and uniformly distributed in the range $(-\pi, \pi)$, after statistical averaging in Eq.(S3) only the terms with $\sigma = l$ survive, i.e. one obtains

$$P_n(t_\alpha) = \sum_{l_1, l_2, ..., l_\alpha} |S_{n,l_1}|^2 \times |S_{l_1,l_2}|^2 \times ... \times |S_{l_{\alpha-1},l_\alpha}|^2 P_{l_\alpha}(t=0) \tag{S5}$$

i.e.

$$P_n(t_\alpha) = \sum_l |S_{n,l}|^2 P_l(t_{\alpha-1}) \tag{S6}$$

Equation (S6) can be written in the equivalent form

$$P_n(t_\alpha) - P_n(t_{\alpha-1}) = \sum_l (|S_{n,l}|^2 - \delta_{n,l}) P_l(t_{\alpha-1}) \tag{S7}$$

which can be viewed as a classical random walk on the lattice, in which $M_{n,l} \equiv |S_{n,l}|^2/\Delta t$ for $l \neq n$ is the transition rate from site l to site n, whereas $-M_{n,n} = (1-|S_{n,n}|^2)/\Delta t$ is the probability loss rate of site n. Assuming a short time interval Δt between successive stochastic phases, from Eq.(S7) it follows that the average unnormalized occupation probabilities satisfy the master equation

$$\frac{dP_n}{dt} = \sum_{l=1}^{N} M_{n,l} P_l(t) \tag{S8}$$

where the elements of the Markov transition matrix M read

$$M_{n,l} = \begin{cases} \frac{|S_{n,l}|^2}{\Delta t} & n \neq l \\ \frac{|S_{n,l}|^2 - 1}{\Delta t} & n = l. \end{cases} \tag{S9}$$

To calculate the explicit form of the Markov transition matrix elements, let us expand the coherent propagation matrix $S = \exp(-iH\Delta t)$ up to the order $\sim \Delta t^2$,

$$S = \exp(-iH\Delta t) \simeq I - i\Delta t H - \frac{1}{2}\Delta t^2 H^2 \tag{S10}$$

so that one obtains

$$|S_{n,l}|^2 = \delta_{n,l} + i\Delta t \delta_{n,l}(H^*_{n,n} - H_{n,n}) + \frac{1}{2}\Delta t^2 \left\{ 2|H_{n,l}|^2 - \delta_{n,l} \sum_q (H_{n,q}H_{q,n} + H^*_{n,q}H^*_{q,n}) \right\} \tag{S11}$$

Finally, from Eqs.(S9) and (S11) one obtains

$$\begin{aligned} M_{n,l} &= 2\delta_{n,l}\mathrm{Im}(H_{n,n}) + \\ &+ \Delta t \left[|H_{n,l}|^2 - \delta_{n,l}\mathrm{Re}\left\{ \sum_q H_{n,q}H_{q,n} \right\} \right]. \end{aligned} \tag{S12}$$

S2. Incoherent critical skin effect

As an additional example of incoherent skin effect in a non-reciprocal system, let us consider a quasi 1D model displaying, under coherent dynamics, the critical skin effect [1]. In this model the spectral properties show a dramatic system size sensitivity near a critical point, in such a way that the OBC energy spectrum in the thermodynamic limit undergoes an abrupt change as a system parameter is slightly varied [1]. Such a behavior is found rather generally when two or more independent NH pumping channels are weakly side-coupled, such as in case of two weakly side-coupled Hatano-Nelson chains (Fig.S1**a**). The Bloch Hamiltonian of this system reads

$$H(k) = \sigma_0 d_0 + t_0 \sigma_x + (V - 2i\delta \sin k)\sigma_z \tag{S13}$$

where $d_0 = 2t_1 \cos k$, σ_l are the Pauli matrices, $(t_1 \pm \delta)$ are the asymmetric hopping amplitudes, with opposite orientation in the two chains (Fig.S1**a**), $\pm V$ their on-site energy offset and t_0 is the side coupling constant. For $t_0 = 0$ the two Hatano-Nelson chains are decoupled, the PBC energy spectrum is composed by two ellipses, which are displaced along the real axis by $2V$. The OBC energy spectrum in the thermodynamic limit is entirely real and composed by two segments inside the two ellipses (Fig.S1**b**); skin eigenstates in each chain localize at opposite edges of the lattice. However, even for an extremely small value of $t_0 \neq 0$, the eigenstates hybridize and, while the PBC energy spectrum is only perturbatively modified, the OBC energy spectrum undergoes a discontinuous change, as shown in Fig.S1**c**. This corresponds to a strong sensitivity of the generalized Brillouin zone on t_0 near the critical point $t_0 = 0$ [1].
Let us now consider the incoherent dynamical regime. In this case the Markov transition matrix in Bloch space associated to the Hamiltonian (S13), calculated using Eq.(S12), reads

$$M(k) = \Delta t(\sigma_0 d_0^{'} + t_0^2 \sigma_x - 4it_1\delta \sin k \sigma_z) \tag{S14}$$

where we have set $d_0^{'} \equiv 2(t_1^2 + \delta^2)\cos k + V^2 - t_0^2 - 2t_1^2 + 2\delta^2$. Clearly, $M(k)$ describes two weakly coupled Hatano-Nelson chains with opposite pumping channels, which are decoupled at $t_0 = 0$, and thus the system displays the critical NH

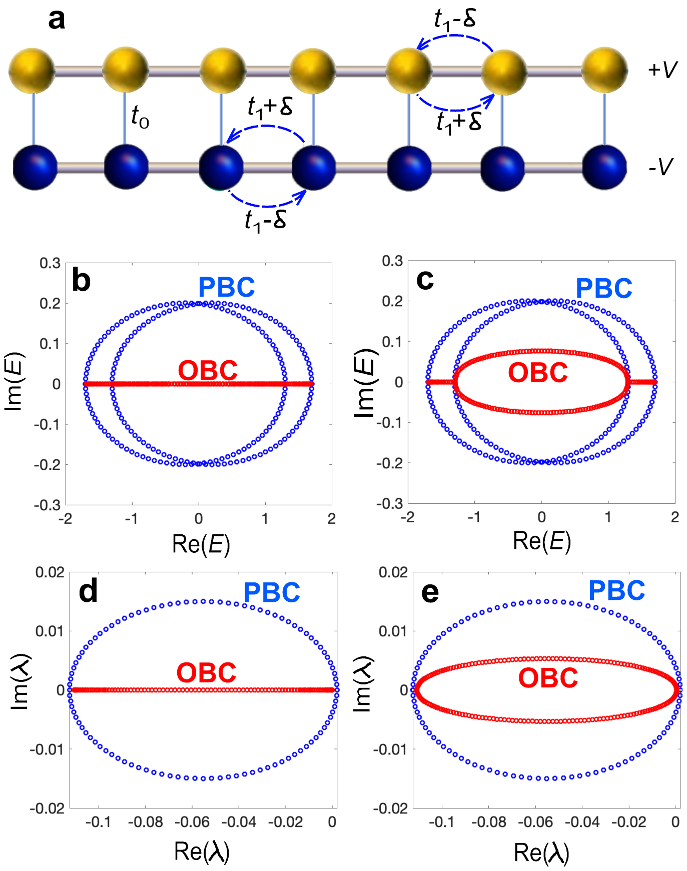

FIG. S1. **Incoherent critical skin effect.** **a** Schematic of two side-coupled Hastano-Nelson chains with opposite NH pumping directions. **b** PBC and OBC energy spectra of the Hamiltonian in the uncoupled case $t_0 = 0$. Other parameter values are $t_1 = 0.75$, $\delta = 0.1$ and $V = 0.2$. Each chain comprises 100 sites. **c** Same as panel **b**, but for two very weakly coupled chains ($t_0 = 0.0001$). Note that, while the PBC energy spectrum remains practically unchanged, the OBC energy spectrum is greatly varied, a clear signature of the critical skin effect. Panels **d,e** display the corresponding loci of eigenvalues of the Markov transition matrix under PBC and OBC (dephasing time interval $\Delta t = 0.05$). Also in the incoherent regime the OBC spectrum of M is strongly sensitive to the side-coupling amplitude t_0.

skin effect, as shown in Fig.S1**d,e**. Note that, since the dephasing introduced by the stochastic phases washes out the on-site potential offset $\pm V$ of the coherent dynamics, under PCB the energy spectrum at $t_0 = 0$ is described by two overlapping ellipses. This means that the critical skin effect persists under incoherent dynamics. However, it should be mentioned that, if the critical skin effect arises in systems with reciprocal hopping [2], the critical skin effect is washed out under incoherent dynamics.

S3. Hamiltonian, Markov transition matrix and drift velocity in coherent and incoherent NH quantum walks

3.1 Coherent quantum walk

The coherent NH quantum walk is defined by the discrete-time coupled equations for the walker amplitudes, in state $|H\rangle$ and $|V\rangle$, at various lattice sites [Eqs.(13) and (14) in the main text]

$$u_n^{(t+1)} = \exp(\gamma)\left(\cos\theta u_{n+1}^{(t)} + i\sin\theta v_{n+1}^{(t)}\right) \tag{S15}$$

$$v_n^{(t+1)} = \exp(-\gamma)\left(i\sin\theta u_{n-1}^{(t)} + \cos\theta v_{n-1}^{(t)}\right). \tag{S16}$$

where θ is the rotation angle entering in the coin operator and γ is the imaginary gauge phase. Owing to discrete spatial translational invariance, the quantum walk dynamics can be described in Bloch space. After letting $u_n^{(t)} =$

$u^{(t)}(k)\exp(ikn)$ and $v_n^{(t)} = v^{(t)}(k)\exp(ikn)$, where k is the Bloch wave number, one obtains $(u^{(t+1)}(k), v^{(t+1)}(k))^T = U_{coh}(k)(u^{(t)}(k), v^{(t)}(k))^T$, where

$$U_{coh}(k) = \begin{pmatrix} \cos\theta\exp(ik+\gamma) & i\sin\theta\exp(ik+\gamma) \\ i\sin\theta\exp(-ik-\gamma) & \cos\theta\exp(-ik-\gamma) \end{pmatrix} \tag{S17}$$

is the one-step propagator in Bloch space. Since $U_{coh}(k)$ is unimodular, we can use the composition law of the SU(2) group and write $U_{coh}(k)$ in terms of the exponential of a Hamiltonian matrix $H(k)$ represented by a Pauli vector, i.e. $U_{coh}(k) = \exp\{-iH(k)\}$ with

$$H(k) = -\frac{a(k)}{\sin a(k)} \begin{pmatrix} \cos\theta\sin(k-i\gamma) & \sin\theta\exp(ik+\gamma) \\ \sin\theta\exp(-ik-\gamma) & -\cos\theta\sin(k-i\gamma) \end{pmatrix} \tag{S18}$$

where we have set

$$a(k) \equiv \text{acos}\left\{\cos\theta\cos(k-i\gamma)\right\}. \tag{S19}$$

The eigenvalues of $H(k)$ are given by $E_\pm(k) = \pm a(k)$ with corresponding eigenvectors

$$\Delta_\pm(k) = \begin{pmatrix} -\sin\theta\exp(ik+\gamma) \\ \cos\theta\sin(k-i\gamma) \pm \sin a(k) \end{pmatrix}. \tag{S20}$$

As discussed in the main manuscript, the Bloch wave number k is real and varies in the Brillouin zone $-\pi \leq k < \pi$ under PBC, whereas for OBC k is complexified and varies in the generalized Brillouin zone $k = q + i\gamma$, with $-\pi \leq q < \pi$. To calculate the drift velocity v_{coh} of any wave packet induced by the imaginary gauge phase γ, let us consider the spectral representation of the solution to Eqs.(S15) and (S16), namely let us write

$$\begin{pmatrix} u_n^{(t)} \\ v_n^{(t)} \end{pmatrix} = \sum_{l=\pm} \int_{-\pi}^{\pi} dk F_l(k)\Delta_l(k)\exp\left\{ikn - iE_l(k)t\right\} \tag{S21}$$

where the spectral amplitudes $F_\pm(k)$ are determined by the initial state of the walker at $t = 0$. The long-time asymptotic behavior of the walker amplitudes $u_n^{(t)}$ and $v_n^{(t)}$ along the space-time line $n = vt$, with v an arbitrary velocity, can be calculated using the steepest-descent method [2] . The drift velocity v_{coh} is the value of v at which the amplitudes asymptotically grow fastest (or decay slowest), and can be calculated from the relation [2]

$$v_{coh} = \text{Re}\left(\frac{dE_\pm}{dk}\right)_{k_0} \tag{S22}$$

where k_0 is the value of k, in the interval $-\pi \leq k < \pi$, at which $\text{Im}(E_\pm(k))$ takes its largest value. Since $E_\pm(k) = \pm a(k)$, from Eq.(S19) it readily follows that $k_0 = \pm\pi/2$ for the two energy branches, yielding after some straightforward calculations

$$v_{coh} = \pm\frac{\cos\theta\cosh\gamma}{\sqrt{1+\cos^2\theta\sinh^2\gamma}} \tag{S23}$$

which is Eq.(16) given in the main text.

3.2 Incoherent quantum walk

The incoherent NH quantum walk under dephasing effects is governed by the following set of discrete-time equations for the non-normalized probabilities $X_n^{(t)} = |u_n^{(t)}|^2$ and $Y_n^{(t)} = |v_n^{(t)}|^2$ to find the walker at lattice site n, in either state $|H\rangle$ or $|V\rangle$ [Eqs.(17) and (18) of the main text]

$$X_n^{(t+1)} = \exp(2\gamma)\left(\cos^2\theta X_{n+1}^{(t)} + \sin^2\theta Y_{n+1}^{(t)}\right) \tag{S24}$$

$$Y_n^{(t+1)} = \exp(-2\gamma)\left(\sin^2\theta X_{n-1}^{(t)} + \cos^2\theta Y_{n-1}^{(t)}\right) \tag{S25}$$

which basically describes a classical random walk. Owing to discrete spatial translational invariance, the random walk dynamics can be described in Bloch space. After letting $X_n^{(t)} = x^{(t)}(k)\exp(ikn)$ and $Y_n^{(t)} = y^{(t)}(k)\exp(ikn)$, where k is the Bloch wave number, one obtains $(x^{(t+1)}(k), y^{(t+1)}(k))^T = U_{inc}(k)(x^{(t)}(k), y^{(t)}(k))^T$, where

$$U_{inc}(k) = \begin{pmatrix} \cos^2\theta \exp(ik+2\gamma) & \sin^2\theta \exp(ik+2\gamma) \\ \sin^2\theta \exp(-ik-2\gamma) & \cos^2\theta \exp(-ik-2\gamma) \end{pmatrix} \tag{S26}$$

is the one-step propagator in Bloch space. Provided that $\det\{U_{inc}(k)\} \neq 0$, the propagator $U_{inc}(k)$ can be written in terms of the exponential of the Markov transition matrix $M(k)$, i.e. $U_{inc}(k) = \exp\{M(k)\}$ with

$$M(k) = \ln\sqrt{2\cos^2\theta - 1}\begin{pmatrix} 1 & 0 \\ 0 & 1 \end{pmatrix} + i\frac{a'(k)}{\sin a'(k)}\frac{1}{\sqrt{2\cos^2\theta - 1}}\begin{pmatrix} \cos^2\theta\sin(k-2i\gamma) & -i\sin^2\theta\exp(ik+2\gamma) \\ -i\sin^2\theta\exp(-ik-2\gamma) & -\cos^2\theta\sin(k-2i\gamma) \end{pmatrix} \tag{S27}$$

where we have set

$$a'(k) \equiv \text{acos}\left\{\frac{\cos^2\theta}{\sqrt{2\cos^2\theta - 1}}\cos(k-2i\gamma)\right\}. \tag{S28}$$

The eigenvalues of $M(k)$ are given by

$$\lambda_\pm(k) = \ln\sqrt{2\cos^2\theta - 1} \pm ia'(k) \tag{S29}$$

with corresponding eigenvectors

$$\Delta_\pm(k) = \begin{pmatrix} i\sin^2\theta\exp(ik+2\gamma) \\ \cos^2\theta\sin(k-2i\gamma) \mp \sqrt{2\cos^2\theta - 1}\sin a(k) \end{pmatrix}. \tag{S30}$$

We note that the elements of the Markov transition matrix $M(k)$ diverge at the exact Hadamard coin angle $\theta = \pi/4$, which is related to the circumstance that at $\theta = \pi/4$ one of the two eigenvalues of the propagator $U_{inc}(k)$ vanishes and $\det\{U_{inc}(k)\} = 0$. However, such a divergence does not have any physical meaning since the propagator $U_{inc}(k)$ is a continuous function of the angle θ, remains finite at $\theta = \pi/4$, and the dynamics is time-discrete. As a matter of fact, the limiting case of Hadamard coin $\theta = \pi/4$ admits of a simple analytical solution in terms of asymmetric binomial distribution, which is presented in Sec.S4 below.
To calculate the drift velocity v_{inc} of any wave packet induced by the imaginary gauge phase γ, let us consider the spectral representation of the solution to Eqs.(S24) and (S25), namely let us write

$$\begin{pmatrix} X_n^{(t)} \\ Y_n^{(t)} \end{pmatrix} = \sum_{l=\pm}\int_{-\pi}^{\pi} dk F_l(k)\Delta_l(k)\exp\{ikn + \lambda_l(k)t\} \tag{S31}$$

where the spectral amplitudes $F_\pm(k)$ are determined by the initial state of the walker at $t = 0$. The long-time asymptotic behavior of the probabilities $X_n^{(t)}$ and $Y_n^{(t)}$ along the space-time line $n = vt$, with v an arbitrary velocity, can be calculated using the steepest-descent method, following the same procedure outlined for the coherent quantum walk case discussed above. The drift velocity v_{inc} is the value of v at which the amplitudes take asymptotically their largest value, and can be calculated from the relation

$$v_{inc} = -\text{Im}\left(\frac{d\lambda_\pm}{dk}\right)_{k_0} \tag{S32}$$

where k_0 is the value of k, in the interval $-\pi \leq k < \pi$, at which $\text{Re}(\lambda_\pm(k))$ takes its largest value. Using Eqs.(S28) and (S29), it readily follows that $k_0 = 0, \pi$ for the two branches, yielding after some straightforward calculations

$$v_{inc} = \frac{\cos^2\theta\sinh(2\gamma)}{\sqrt{\sin^4\theta + \cos^4\theta\sinh^2(2\gamma)}} \tag{S33}$$

which is Eq.(21) given in the main text.

S4. Incoherent NH quantum walk for the Hadamard coin

For the Hadamard coin ($\theta=\pi/4$), the incoherent NH quantum walk takes the form

$$X_n^{(t+1)} = \frac{1}{2}\exp(2\gamma)\left(X_{n+1}^{(t)}+Y_{n+1}^{(t)}\right) \quad \text{(S34)}$$

$$Y_n^{(t+1)} = \frac{1}{2}\exp(-2\gamma)\left(X_{n-1}^{(t)}+Y_{n-1}^{(t)}\right) \quad \text{(S35)}$$

which can be readily solved in Bloch (Fourier) space. The one-step propagator $U_{inc}(k)$ reads [Eq.(S26) with $\theta=\pi/4$]

$$U_{inc}(k)=\frac{1}{2}\left(\begin{array}{cc} \exp(ik+2\gamma) & \exp(ik+2\gamma) \\ \exp(-ik-2\gamma) & \exp(-ik-2\gamma) \end{array}\right) \quad \text{(S36)}$$

so that the most general to Eqs.(S34) and (S35) admits of the spectral representation

$$\left(\begin{array}{c} X_n^{(t)} \\ Y_n^{(t)} \end{array}\right)=\int_{-\pi}^{\pi} dk U_{inc}^t(k)\left(\begin{array}{c} A_X(k) \\ A_Y(k) \end{array}\right)\exp(ikn) \quad \text{(S37)}$$

where the spectral amplitudes $A_X(k)$, $A_Y(k)$ are determined by the initial state $X_n^{(0)}$, $Y_n^{(0)}$ of the walker according to

$$A_X(k)=\frac{1}{2\pi}\sum_n X_n^{(0)}\exp(-ikn)\ , \ \ A_Y(k)=\frac{1}{2\pi}\sum_n Y_n^{(0)}\exp(-ikn) \quad \text{(S38)}$$

The tth power of the propagator, $U_{inc}^t(k)$, can be readily computed after matrix diagonalization and reads explicitly

$$U_{inc}^t(k)=\cos^{t-1}(k-2i\gamma)U_{inc}(k). \quad \text{(S39)}$$

From Eqs.(S37), (S38) and (S39) one obtains for $t=1,2,3,...$

$$X_n^{(t)}=\frac{1}{4\pi}\sum_l\int_{-\pi}^{\pi} dk\cos^{t-1}(k-2i\gamma)\exp(ik+ikn-ikl+2\gamma)\left(X_l^{(0)}+Y_l^{(0)}\right) \quad \text{(S40)}$$

$$Y_n^{(t)}=\frac{1}{4\pi}\sum_l\int_{-\pi}^{\pi} dk\cos^{t-1}(k-2i\gamma)\exp(-ik+ikn-ikl-2\gamma)\left(X_l^{(0)}+Y_l^{(0)}\right) \quad \text{(S41)}$$

so that the probability $P_n^{(t)}=X_n^{(t)}+Y_n^{(t)}$ to find the walker at lattice site n, regardless of V or H internal state, reads

$$P_n^{(t)}=\frac{1}{2\pi}\sum_l\int_{-\pi}^{\pi} dk\cos^{t}(k-2i\gamma)\exp(ikn-ikl)P_l^{(0)} \quad \text{(S42)}$$

Taking into account that

$$\cos^t(k-2i\gamma)=\frac{1}{2^t}\sum_{\sigma=0}^{t}\left(\begin{array}{c} t \\ \sigma \end{array}\right)\exp[(ik+2\gamma)(2\sigma-t)] \quad \text{(S43)}$$

one finally obtains for $P_n^{(t)}$ the analytical form in terms of binomial coefficients

$$P_n^{(t)}=\frac{1}{2^t}\sum_{\sigma=0}^{t}\left(\begin{array}{c} t \\ \sigma \end{array}\right)\exp[2\gamma(2\sigma-t)]P_{2\sigma-t+n}^{(0)} \quad \text{(S44)}$$

For example, if the walker at initial time is at site $n=0$, $P_l^{(0)}=\delta_{l,0}$, one has

$$P_n^{(t)}=\left\{\begin{array}{cc} 0 & \text{if } \ |n|>t \ \text{ or } \ (t-n) \ \text{ odd} \\ \frac{1}{2^t}\exp(-2\gamma n)\left(\begin{array}{c} t \\ (t-n)/2 \end{array}\right) & \text{otherwise} \end{array}\right. . \quad \text{(S45)}$$

In the Hermitian limit $\gamma=0$, the distribution is symmetric around $n=0$, the total probability $\sum_n P_n^{(t)}=1$ is conserved, and the above result reduces to a classical random walk on a line with equal probability to move on left/right

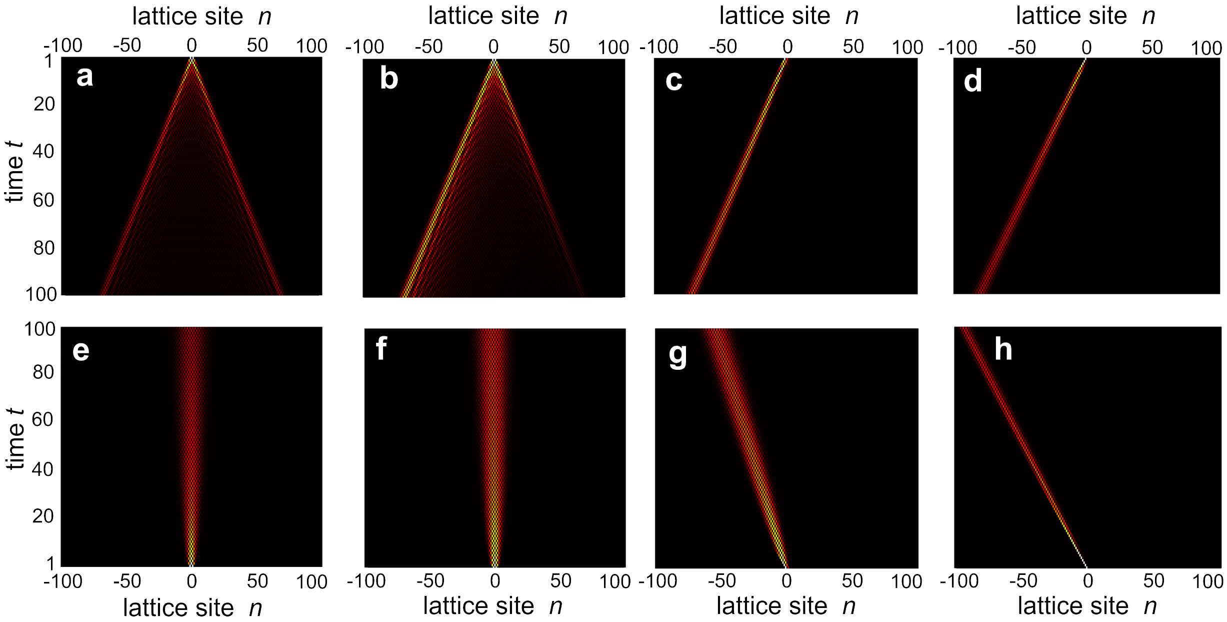

FIG. S2. **Coherent versus incoherent spreading dynamics for Hadamard coin**. **a-d** Evolution of normalized occupation probability of the walker at various lattice sites for the initial state $|\psi(0)\rangle = (1/\sqrt{2})(|H\rangle\langle H| + |V\rangle\langle V|) \otimes |n\rangle\langle n|$, $\theta = \pi/4$ (Hadamard coin), and for increasing values of the imaginary gauge phase γ. **a** $\gamma = 0$ (Hermitian quantum walk); **b** $\gamma = 0.01$; **c** $\gamma = 0.3$; **d** $\gamma = 0.8$. **e-h**: same as **a-d** but for the incoherent quantum walk.

.

sides [3]. The distribution probability displays diffusive spreading, which is characteristic of a classical random walk [3]. In the NH case $\gamma \neq 0$, the probability distribution is asymmetric, the total probability is not conserved and the peak distribution drifts in time at a speed which is given by Eq.(S33) with $\theta = \pi/4$, i.e. $v_{inc} = \pm\tanh(2\gamma)$, which vanishes in the weak NH limit $\gamma \to 0$. We note that, after normalization of $P_n^{(t)}$ at each time step, for $\gamma \neq 0$ we basically have a classical random walk with asymmetric left/right hopping probabilities, which explains the drift dynamics in the bulk and accumulation of excitation at one edge of the lattice under OBC.

It is worth comparing the spreading dynamics in the incoherent versus coherent regimes at the Hadamard angle, as the imaginary gauge phase is increased above zero, which is depicted in Fig.S2. Note that in the Hermitian limit $\gamma = 0$ the spreading is ballistic in the coherent regime and occurs along the two space-time lines $v_{coh} = \pm\cos(\theta) = \pm\sqrt{2}/2$ (Fog.S2**a**). As γ is slightly increased above zero, one of the two peaks dominates over the other one, and the excitation asymptotically undergoes unidirectional drift along the lattice at the speed close to $\sqrt{2}/2$ (Fig.S2**b**). The drift velocity increases as γ is increased (Fig.S2**c,d**) to reach the upper limit $v_{coh} \simeq 1$ in the large γ limit. Conversely, in the incoherent regime the spreading dynamics in the Hermitian limit is diffusive with a single main peak along the space-time line $n = 0$ (Fig.S2**e**). In this case, as γ is slightly increased above zero, the probability distribution is only slightly affected, acquiring a very small drift velocity (Fig.S2**f**). However, as γ is further increased, according to the theoretical analysis and Fig.4**f** of the main manuscript the drift velocity in the incoherent regime overcomes the one in the coherent regime (Fig.S2**h**).

[1] Li, L., Lee, C.H., Mu, S. & Gong, J. Critical non-Hermitian skin effect. *Nature Commun.* **11**, 5491 (2020).
[2] Longhi, S. Probing non-Hermitian skin effect and non-Bloch phase transitions. *Phys. Rev. Res.* **1**, 023013 (2019).
[3] Venegas-Andraca, S.E. Quantum walks: a comprehensive review. *Quantum Inf. Process* **11**, 1015 (2012).
